# Computational Photosynthesis (ComPhot): Simulation-Based Learning Platform to Study Photosynthesis

**DOI:** 10.1093/plcell/koae101

**Published:** 2024-05-31

**Authors:** Sarah Philipps, Tobias Pfennig, Elouën Corvest, Marvin van Aalst, Lisa Fürtauer, Anna Matuszyńska

**Affiliations:** Computational Life Science, Department of Biology, RWTH Aachen University, Aachen, Germany; Computational Life Science, Department of Biology, RWTH Aachen University, Aachen, Germany; Computational Life Science, Department of Biology, RWTH Aachen University, Aachen, Germany; Institute for Quantitative and Theoretical Biology, Heinrich Heine University Düsseldorf, Germany; Plant Molecular Systems Biology, Department of Biology, RWTH Aachen University, Aachen, Germany; Computational Life Science, Department of Biology, RWTH Aachen University, Aachen, Germany

## Abstract

Studies show the advantage of active versus passive learning formats in delivering complicated concepts (Minocha and Clarke, 2009; Pluta *et al.*, 2013). Hence, interactive teaching tools are not only more often positively evaluated by students but also contribute to better life-long teaching outcomes (Ang *et al.*, 2021). Following this evidence, we created ComPhot, a stand-alone learning platform for motivated students and researchers. It guides the user in studying photosynthesis as a well-known biological process with the support of a computational model. ComPhot is a no-code, easy-to-use tool to lower the entry bar for starting the journey across computational biology and to provide insights into how photosynthesis and modeling photosynthesis work. This user-friendly interactive **teaching platform** can be used individually or to support teachers following a syllabus in biology, to include the concept of computational biology or mathematics, to show the possible field of application of mathematics to biology.

ComPhot introduces and explains the biochemical background of our simulated system and how to translate it into mathematical terms. We provide **diverse teaching materials** that include text, guiding questions, videos, and, most importantly, simulations. Within our simulators, users can perform computational photosynthesis modeling in their browser by simply setting and manipulating slider bars. Our comprehensive approach conveys fundamental insights into photosynthesis, photoprotection, and fluorescence measurements and empowers users to devise their own *in silico* experiments by varying light conditions or designing synthetic strains. This tool acts as a stepping stone, fostering engagement and understanding while propelling research and innovation in photosynthesis. Although this guide has been written in English, we are proud to release the tool in four of the developers’ languages to expand the audience: **English, German, French,** and **Polish**.

RECOMMENDED CITATION STYLE: **Phillips S., Prenning T, Corvest E, van Aalst, M, Fürtauer L.** Computational photosynthesis (ComPhot): Simulation-based learning platform to study photosynthesis. Teaching Tools in Plant Biology: Lecture Notes. *The Plant Cell*.

## Supplementary Material

koae101_Supplementary_Data

