# Supplementary material for: Computational Photosynthesis (ComPhot): Simulation-Based Learning Platform to Study Photosynthesis
Source: Plant Cell. 2024 May 31;37(6):koae101. doi: 10.1093/plcell/koae101 (PMC12214869; doi:10.1093/plcell/koae101)
Supplement: koae101_Supplementary_Data [file koae101_Supplementary_Data.zip › TTPB46TeachingGuide.pdf]

Computational Photosynthesis (ComPhot): Simulation-Based Learning Platform to Study Photosynthesis

By Sarah Philipps, Tobias Pfennig, Elouën Corvest, Marvin van Aalst, Lisa Fürtauer, and Anna Matuszyńska

OVERVIEW

Studies show the advantage of active versus passive learning formats in delivering complicated concepts (Minocha and Clarke, 2009; Pluta *et al.*, 2013). Hence, interactive teaching tools are not only more often positively evaluated by students but also contribute to better life-long teaching outcomes (Ang *et al.*, 2021). Following this evidence, we created ComPhot, a stand-alone learning platform for motivated students and researchers. It guides the user in studying photosynthesis as a well-known biological process with the support of a computational model. ComPhot is a no-code, easy-to-use tool to lower the entry bar for starting the journey across computational biology and to provide insights into how photosynthesis and modeling photosynthesis work. This user-friendly interactive **teaching platform** can be used individually or to support teachers following a syllabus in biology, to include the concept of computational biology or mathematics, to show the possible field of application of mathematics to biology.

ComPhot introduces and explains the biochemical background of our simulated system and how to translate it into mathematical terms. We provide **diverse teaching materials** that include text, guiding questions, videos, and, most importantly, simulations. Within our simulators, users can perform computational photosynthesis modeling in their browser by simply setting and manipulating slider bars. Our comprehensive approach conveys fundamental insights into photosynthesis, photoprotection, and fluorescence measurements and empowers users to devise their own *in silico* experiments by varying light conditions or designing synthetic strains. This tool acts as a stepping stone, fostering engagement and understanding while propelling research and innovation in photosynthesis. Although this guide has been written in English, we are proud to release the tool in four of the developers' languages to expand the audience: **English, German, French,** and **Polish**.

USING THE PLATFORM

The learning platform is an interactive website that is available online at https://comphot-biotool.streamlit.app/ or can be downloaded from https://github.com/AnnaMatuszynska/biotool-photosynthesis and run locally (offline). We used the Python programming language to build the website in the open-source app frame Streamlit and implement reactive models to the user input. All that is necessary to use ComPhot optimally is a browser with an active internet connection. We recommend using a laptop or personal computer; however, mobile devices are also supported. More experienced users can also download the source files to run the website locally and offline. In this case, a computer capable of installing and running Python packages is necessary.

The website is split into multiple pages that cover different topics, from the biological background of photosynthesis to the simulation. It is recommended to follow them sequentially. The first page, ***Start***, explains in detail how ComPhot is used. To navigate between the pages, the user can click the arrow in the top left corner to open a navigation panel. At the top of this panel, the user can select any of the platform's pages by clicking on the name. The navigation panel also allows selecting the different languages and two website versions for different target groups: ***4Bio*** and ***4Math***. In the panel, the user can also activate transcripts for the YouTube videos on the website. The content of the **4Bio** version is designed for students with solid knowledge and/or interest in biology; hence, it is advised for use by

biology teachers/lecturers. Alternatively, we suggest teaching students with solid foundations in engineering, mathematics, or informatics, following the content of version **4Math**.

The five main parts of the website consist of introductory teaching material on i) **Photosynthesis**, ii) **Measuring Method**, and iii) **Computational models** and interactive subpages: iv) **Experiments *in silico***, and v) **Plant Light Memory**. Each subpage contains the top information about the specific learning objectives and required knowledge to appreciate the content. Each page can be studied during

4Bio VS 4 Math

The 4Bio version explains mathematical modeling and showcases how computational models support biological research. It explores the existing models in more detail by allowing them to change additional parameters and simulate more complex experiments. Meanwhile, the 4Math version provides further information on the model construction and implementation in Python. Here, we also include our preferred way of model implementation by using our in-house developed package that streamlines the development of computational models capturing dynamic changes over time.

Regardless of the user's background, after completing all steps, they will have learned about state-of-the-art measurements in plant sciences, the ”short-term light memory” of plants, some examples of mathematical models, and the effects changes can have on the modeled photosynthetic system. We hope to inspire the users to look at the model standing behind the simulations and maybe start their journey of learning how to program with this excellent Python course published previously: Plants & Python (vanBuren *et al.*, 2022).

TEACHING GOAL

Our goal is to leave the reader informed about the interplay between experimental and computational biology. Students who approach the tool from a more biology-oriented perspective should understand how models are created and used for in-depth interpretation of biological systems. Specifically, we want to present a molecular mechanism of photoprotection based on the de-epoxidation of a xanthophyll pigment (violaxanthin). Students with more technical/mathematical training are introduced to how their skills can be utilized for environmental research, mainly modeling photosynthesis. By examining graphs made with computer models, our users learn about the impact of light intensity on photosynthesis and delve into the subject of a lesser-known phenomenon of plant short-term memory. It is desired that the users will be motivated to pursue further research in computational modeling. We are confident that we will thoroughly introduce the captivating realm of (computational) plant biology through this website.

LEARNING OBJECTIVES

Students who investigated the platform in detail should be able to:

- Explain what models are and how they can be used in biology,
- Name some classical examples of models,
- Summarize how non-photochemical quenching works and how it is used in light protection,
- Summarize how fluorescence can be used to measure photosynthesis and
- Produce photosynthesis simulations and explain their biological meaning.

ADDITIONAL RESOURCES

For highly motivated students, additional references are provided on the website. These include:

Bellasio, C., Quirk, J., Buckley, T. N., & Beerling, D. J. (2017). A Dynamic Hydro-Mechanical and Biochemical Model of Stomatal Conductance for C4 Photosynthesis. Plant Physiology, 175(1), 104–119. https://doi.org/10.1104/pp.17.00666

Bellasio, C. (2019). A generalised dynamic model of leaf-level C3 photosynthesis combining light and dark reactions with stomatal behaviour. Photosynthesis Research, 141(1), 99–118. https://doi.org/10.1007/s11120-018-0601-1

Brooks, M. D., & Niyogi, K. K. (2011). Use of a pulse-amplitude modulated chlorophyll fluorometer to study the efficiency of photosynthesis in Arabidopsis plants. Chloroplast Research in Arabidopsis: Methods and Protocols, Volume II, 299-310. https://link.springer.com/protocol/10.1007/978-1-61779-237-3_16

Cook, J., Oreskes, N., Doran, P. T., Anderegg, W. R. L., Verheggen, B., Maibach, E. W., Carlton, J. S., Lewandowsky, S., Skuce, A. G., Green, S. A., Nuccitelli, D., Jacobs, P., Richardson, M., Winkler, B., Painting, R., & Rice, K. (2016). Consensus on consensus: A synthesis of consensus estimates on human-caused global warming. Environmental Research Letters, 11(4), 048002. https://doi.org/10.1088/1748-9326/11/4/048002

Evans, J., & Von Caemmerer, S. (2012). Temperature response of carbon isotope discrimination and mesophyll conductance in tobacco. Plant, Cell & Environment, 36(4), 745–756. https://doi.org/10.1111/j.1365-3040.2012.02591.x

Farquhar, G. D., von Caemmerer, S., & Berry, J. A. (1980). A biochemical model of photosynthetic CO_2 assimilation in leaves of C3 species. Planta, 149(1), 78–90. https://doi.org/10.1007/BF00386231

Matuszyńska, A., Heidari, S., Jahns, P., & Ebenhöh, O. (2016). A mathematical model of non-photochemical quenching to study short-term light memory in plants. Biochimica et Biophysica Acta (BBA) - Bioenergetics, 1857(12), 1860–1869. https://doi.org/10.1016/j.bbabio.2016.09.003
The original model used in the sections on experiments *in silico* and Plant Memory.

Nies, T., Niu, Y., Ebenhöh, O., Matsubara, S., & Matuszyńska, A. (2021). Chlorophyll fluorescence: How the quality of information about PAM instrument parameters may affect our research (p. 2021.05.12.443801). bioRxiv. https://doi.org/10.1101/2021.05.12.443801

Price, G. D., Badger, M. R., & Von Caemmerer, S. (2010). The prospect of using cyanobacterial bicarbonate transporters to improve leaf photosynthesis in C3 crop plants. Plant Physiology, 155(1), 20–26. https://doi.org/10.1104/pp.110.164681

Taiz, L., Zeiger, E., Møller, I. M., & Murphy, A. S. (2018). Fundamentals of plant physiology (First edition). Published in the United States of America by Oxford University Press.

Von Caemmerer, S. (2013). Steady-state models of photosynthesis. Plant, Cell & Environment, 36(9), 1617–1630. https://doi.org/10.1111/pce.12098

Yin, X., Van Oijen, M., & Schapendonk, A. H. C. M. (2004). Extension of a biochemical model for the generalized stoichiometry of electron transport limited C3 photosynthesis. Plant, Cell & Environment, 27(10), 1211–1222. https://doi.org/10.1111/j.1365-3040.2004.01224.x

Zhu, X.-G., Wang, Y., Ort, D. R., & Long, S. P. (2013). e-photosynthesis: A comprehensive dynamic mechanistic model of C3 photosynthesis: from light capture to sucrose synthesis. Plant, Cell & Environment, 36(9), 1711–1727. https://doi.org/10.1111/pce.12025

Zhu, X.-G., Govindjee, Baker, N. R., deSturler, E., Ort, D. R., & Long, S. P. (2005). Chlorophyll a fluorescence induction kinetics in leaves predicted from a model describing each discrete step of excitation energy and electron transfer associated with Photosystem II. Planta, 223(1), 114–133. https://doi.org/10.1007/s00425-005-0064-4

Zhu, X.-G., De Sturler, E., & Long, S. P. (2007). Optimizing the Distribution of Resources between Enzymes of Carbon Metabolism Can Dramatically Increase Photosynthetic Rate: A Numerical Simulation Using an Evolutionary Algorithm. Plant Physiology, 145(2), 513–526. https://doi.org/10.1104/pp.107.103713

Also, students that are interested in programming and not only using sliders on the website to see the end effect of modeling can use another Teaching Tool: *Plants and Python*, a Python course for Biologists:

VanBuren, R., Rougon-Cardoso, A., Amézquita, E.J., Coss-Navarrete, E.L., Espinosa-Jaime, A., Gonzalez-Iturbe, O.A., Luckie-Duque, A.C., Mendoza-Galindo, E., Pardo, J., Rodríguez-Guerrero, G., Rosiles-Loeza, P.Y., Vásquez-Cruz, M., Fernandez-Valverde, S.L., Hernández-Hernández, T., Palande, S., and Chitwood, D.H. (2022) "Plants & Python: A series of lessons in coding, plant biology, computation, and bioinformatics. Teaching Tools in Plant Biology: Lecture Notes." The Plant Cell, 34(7), e1. https://doi.org/10.1093/plcell/koac187 Official Website: https://plantsandpython.github.io/PlantsAndPython

Sarah Philipps
sarah.philipps@rwth-aachen.de
Computational Life Science, Department of Biology, RWTH Aachen University, Aachen, Germany

Tobias Pfennig
tobias.pfennig@rwth-aachen.de
ORCID: 0000-0002-3825-2778
Computational Life Science, Department of Biology, RWTH Aachen University, Aachen, Germany

Elouën Corvest
elouen.corvest@rwth-aachen.de
ORCID: 0009-0006-3796-9343
Computational Life Science, Department of Biology, RWTH Aachen University, Aachen, Germany

Marvin van Aalst
marvin.van.aalst@hhu.de
ORCID: 0000-0002-7434-0249
Institute for Quantitative and Theoretical Biology, Heinrich Heine University Düsseldorf, Germany

Lisa Fürtauer
lisa.fuertauer@bio3.rwth-aachen.de
ORCID: 0000-0001-5248-4105
Plant Molecular Systems Biology, Department of Biology, RWTH Aachen University, Aachen, Germany

Anna Matuszyńska
anna.matuszynska@cpbl.rwth-aachen.de
ORCID: 0000-0003-0882-6088
Computational Life Science, Department of Biology, RWTH Aachen University, Aachen, Germany

REFERENCES

Ang, K.C.S., Afzal, F., and Crawford, L.H. (2021) "Transitioning from passive to active learning: Preparing future project leaders". In: Project Leadership and Society 2, p. 100016. doi: 10.1016/j.plas.2021.100016

Minocha, S. and Clarke, T. (2009) “Role of social software tools in education: a literature review”. In: Education + Training 51. doi:10.1108/00400910910987174

Pluta, W.J., Richards, B.F., and Mutnick, A. (2013) “PBL and Beyond: Trends in Collaborative Learning”. In: Teaching and Learning in Medicine 25.sup1. PMID: 24246112, S9–S16. doi: 10.1080/10401334.2013.842917.

VanBuren, R., Rougon-Cardoso, A., Amézquita, E.J., Coss-Navarrete, E.L., Espinosa-Jaime, A., Gonzalez-Iturbe, O.A., Luckie-Duque, A.C., Mendoza-Galindo, E., Pardo, J., Rodríguez-Guerrero, G., Rosiles-Loeza, P.Y., Vásquez-Cruz, M., Fernandez-Valverde, S.L., Hernández-Hernández, T., Palande, S., and Chitwood, D.H. (2022) “Plants & Python: A series of lessons in coding, plant biology, computation, and bioinformatics. Teaching Tools in Plant Biology: Lecture Notes.” The Plant Cell, 34(7), e1. https://doi.org/10.1093/plcell/koac187
