# Supplementary material for: Computational Photosynthesis (ComPhot): Simulation-Based Learning Platform to Study Photosynthesis
Source: Plant Cell. 2024 May 31;37(6):koae101. doi: 10.1093/plcell/koae101 (PMC12214869; doi:10.1093/plcell/koae101)
Supplement: koae101_Supplementary_Data [file koae101_Supplementary_Data.zip › TTPB46LectureNotes.pdf]

Computational Photosynthesis (ComPhot): Simulation-Based Learning Platform to Study Photosynthesis

By Sarah Philipps, Tobias Pfennig, Elouën Corvest, Marvin van Aalst, Lisa Fürtauer, and Anna Matuszyńska

ComPhot is a no-code, easy-to-use tool to lower the entry bar for starting the journey across computational biology and to provide insights into how photosynthesis and modeling photosynthesis work. The complete tool is ready to use at https://comphot-biotool.streamlit.app/ or can be run locally offline (see the Teaching Guide). This user-friendly interactive teaching platform introduces and explains the biochemical background of our simulated system and how to translate it using mathematical terms. Within our simulators, the user can perform computational modeling of photosynthesis in their browser by simply setting and manipulating slider bars. Our comprehensive approach conveys fundamental insights into photosynthesis, photoprotection, and fluorescence measurements. It empowers users to devise their own *in silico* experiments by varying light conditions or designing synthetic strains. This tool acts as a stepping stone, fostering engagement and understanding while propelling research and innovation in the realm of photosynthesis. The accompanying Teaching Guide explains our motivation for creating such a platform and describes how to use it online. Although the guide has been written in English, we are proud to release the tool in four of the developers' languages to expand the audience: English, German, Polish, and French.

MOTIVATION

Photosynthesis is arguably the most important biochemical process that drives life on Earth. While its basics are taught in early biology education, photosynthesis research has progressed for more than 300 years. With new technological advances, we can get an understanding down to the level of molecules and atoms. Beyond scientific curiosity, the urgency of addressing climate change and food shortages has propelled the exploration of photosynthesis optimization. In response, scientific communities strive to decipher its intricate mechanisms to amplify crop yield. However, the complex and interwoven nature of photosynthesis presents challenges akin to searching for a needle in a haystack. In this context, computational models emerge as essential tools. They unravel system responses, uncover hidden patterns, and identify potential avenues for intervention. Yet, mastery of biology and computer science is often a challenge. As the computational branch in biology is rapidly growing and so much exciting work is going on, it can be incredibly overwhelming for newcomers. In parallel, those who receive solid computational or mathematical training have often not been exposed enough to current problems in life science to understand the transferability and value of their skills. To bridge this gap, we created this tool, hoping to inspire more people to connect their interest in biology with mathematical and programming skills.

WHY PHOTOSYNTHESIS? - ENVIRONMENTAL CONTEXT

Photosynthesis is one of the most fundamental processes for life we know on Earth. Life depends on the captured photosynthetic energy and its conversion to energy-rich organic molecules. Besides providing bioenergy, food, and fuel, we could not breathe without the by-product oxygen. Due to anthropogenic climate change, agricultural productivity growth has been reported slowing down globally. Estimates predict between the years 2010 and 2050, we should increase the production of major crops by around 50% to

keep up with the ever-growing number of people [1], with a rise of production of 77% in developing countries and 24% in developed countries [2]. To meet principal goals limiting climate change, like the one set by the Paris Agreement to limit global warming to well below 2℃, preferably to 1.5℃, we need research to be fast. Simultaneously, the area where we can plant crops is limited, and more land is currently cultivated at the cost of nature, often rainforests, which is all but sustainable [3]. Due to their sessile lifestyle, climate change is known to impact massively plant performance due to changed abiotic factors like salinity, flooding, drought, heat, and cold. The plants' adaptation to changed environmental conditions is highly dynamic and needs to be fast to ensure survival and yield. These immediate responses allow plants to finally adapt to a new metabolic homeostasis called acclimation.

Hence, the most viable solution is improving crops, where tweaking photosynthesis may provide innovative strategies for increasing product yield. An excellent, and not an isolated, example of a success story is the collaborative research between scientists at the University of Illinois and the University of California Berkeley, USA, who showed that they could improve field crop production by 15 % (!) when speeding up a particular reaction's recovery process (e.g., in tobacco [4] or soybean [5]). Furthermore, connected downstream metabolism needs to be considered for improvements in food production. Also, combined modeling approaches can help to predict and understand plant performances.

Undoubtedly, with our developed and presented Computational Photosynthesis tool, we want to equip you with the tools needed to contribute to the improvement of *in silico,* a.k.a. ”in the computer” models. Computers are much faster at maths than any human can work in a lab and require much fewer resources. Computational biologists try to use this as an advantage by creating mathematical models of different parts of plants or other interesting systems and then predicting the outcomes of modifications the experimentalist could try. This cheap and fast feedback loop helps experimentalists decide which experiments might be worth their money and effort. As a result, efficient teamwork saves time and resources.

USING THE PLATFORM

As a user, you can work on the material at your speed and intensity. The whole website is designed with two target groups in mind. Students with strong knowledge and/or interest in biology are encouraged to explore the **4Bio** version. Students with solid engineering, mathematics, or informatics foundations should explore **4Math**. While the 4Bio version focuses more on exploring the existing models by allowing the user to change more parameters to showcase how computational models support biological research, the 4Math version provides additional information on the model construction and implementation in the Python programming language, including our in-house developed package that streamlines the development of computational models that intend to capture the dynamic changes over the time.

Regardless of your background, after completing all the steps, all users are expected to learn about state-of-the-art measurements in plant sciences, the ”short-term light memory” of plants, and know some examples of mathematical models, even beyond presented models of photosynthesis. This includes classical models used even for tracking the spread of COVID-19 (!). We hope to inspire the users to look at the model standing behind the simulations and maybe even start their journey of learning how to program with this nice Python course published previously: Plants & Python [6].

There are five main parts of the website consisting of introductory material on i) Photosynthesis, ii) Measuring Method and interactive subpages: iii) Computational models, iv) Experiments *in silico*, and v) Plant Light Memory. Each subpage contains the top information about the specific learning objectives and required knowledge to appreciate the content. For all scientific parts, the scientific literature is given within the pages.

CONTENT OF THE SUBPAGES
Each subpage includes lessons on a distinct topic and it is recommended to follow them sequentially. While creating models is not always easy, interacting with and learning about them should be. We hope this tool provides just that: a means for everyone interested to learn about photosynthesis and to experience the joy of modeling. The sub-page **Computational Models** introduces the basic concepts of mathematical models, how to create such a model, and why mathematics is a way to translate processes and problems into general statements by using mathematical descriptions and equations. Again, we provide a custom video where we have summarized the process of model development. In version 4Math, we additionally include examples of source code showing how to translate concepts not only to equations but also a step further to an executable program.

Before diving into photosynthetic models you will learn about the concept of differential equations based models using a classical model of infection spread. The model's initial conditions and parameter values can be conveniently changed using sliders, and new calculations can be visualized on a graph. To show how models can differ in size, format and methodology, you can study the three selected computational models of photosynthesis that we briefly described in terms of their structure, strengths, and differences. In the subpage ***Experiments in silico*** we introduce you to a previously published, highly simplified mathematical model of photosynthesis, created with a focus on understanding the photoprotection of a model organism, *Arabidopsis thaliana* [7]. If you do not know what a model organism is, our platform will introduce you to the importance of model organisms in biological research, too! The user can explore model simulations using handy sliders to pass input to the model and perform simulations online, with the advanced mode offering more options and readouts. Once more, this is supported by a corresponding YouTube video, which shows how to use the sliders and interpret the graphs. In the subpage **Plant Light Memory** we connect all information from lessons included in the previous subpages allowing you to perform your experiments with various light intensities and observing how changing parameters can improve plants response to the stress coming from too high intensities!

AVAILABILITY
The website is available and directly usable online at https://comphot-biotool.streamlit.app/. Additionally, all source code can be downloaded from GitHub at https://github.com/AnnaMatuszynska/biotool-photosynthesis and run (offline) locally. For information on downloading and running the files, please refer to the README.md file on GitHub.

FUNDING
This work was funded by the Deutsche Forschungsgemeinschaft (DFG) - Projektnummer 507704013 (AM), DFG Germany´s Excellence Strategy – EXC-2048/1 – project ID 390686111 (TP, AM), DFG Research Grant - project ID 420069095 (SP, AM), and EU's Horizon 2020 research and innovation programme under the Grant Agreement 862087 (M.v.A.).

ACKNOWLEDGMENT
We would like to thank Joost van Dongen for the helpful discussion at the beginning of the project, Dimitri Neb and Ulrich Schaffrath for the support in creating the video on PAM fluorescence, Lutz Kupferschlaeger for the professional video edit.

Thank you for your interest in learning with us, and best wishes in your computational endeavors.

Sarah Philipps
sarah.philipps@rwth-aachen.de
Computational Life Science, Department of Biology, RWTH Aachen University, Aachen, Germany

Tobias Pfennig
tobias.pfennig@rwth-aachen.de
ORCID: 0000-0002-3825-2778
Computational Life Science, Department of Biology, RWTH Aachen University, Aachen, Germany

Elouën Corvest
elouen.corvest@rwth-aachen.de
ORCID: 0009-0006-3796-9343
Computational Life Science, Department of Biology, RWTH Aachen University, Aachen, Germany

Marvin van Aalst
marvin.van.aalst@hhu.de
ORCID: 0000-0002-7434-0249
Institute for Quantitative and Theoretical Biology, Heinrich Heine University Düsseldorf, Germany

Lisa Fürtauer
lisa.fuertauer@bio3.rwth-aachen.de
ORCID: 0000-0001-5248-4105
Plant Molecular Systems Biology, Department of Biology, RWTH Aachen University, Aachen, Germany

Anna Matuszyńska
anna.matuszynska@cpbl.rwth-aachen.de
ORCID: 0000-0003-0882-6088
Computational Life Science, Department of Biology, RWTH Aachen University, Aachen, Germany

REFERENCES

[1] Michiel van Dijk et al. "A Meta-Analysis of Projected Global Food Demand and Population at Risk of Hunger for the Period 2010–2050". In: Nature Food 2021 2:7 (2021), pp. 494–501. doi: 10.1038/s43016-021-00322-9

[2] Nikos Alexandratos and Jelle Bruinsma. "World agriculture towards 2030/2050: the 2012 revision". In: ESA Working Papers 12-03 (2012). doi: https://doi.org/10.22004/ag.econ.288998.

[3] Claudia M. Viana et al. "Agricultural land systems importance for supporting food security and sustainable development goals: A systematic review". In: Science of The Total Environment 806 (2022), p. 150718. doi: https://doi.org/10.1016/j.scitotenv.2021.150718.

[4] Johannes Kromdijk et al. "Improving photosynthesis and crop productivity by accelerating recovery from photoprotection". In: Science 354.6314 (2016), pp. 857–861. doi: 10.1126/science.aai8878.

[5] Amanda P. De Souza et al. "Soybean photosynthesis and crop yield are improved by accelerating recovery from photoprotection". In: Science 377.6608 (2022), pp. 851–854. doi: 10.1126/science.adc9831.

[6] "Plants & Python: A series of lessons in coding, plant biology, computation, and bioinformatics". In: The Plant Cell 34.7 (2022), e1–e1. doi: 10.1093/plcel /koac187.

[7] Anna Matuszyńska et al. "A mathematical model of non-photochemical quenching to study short-term light memory in plants". In: Biochimica et Biophysica Acta (BBA) - Bioenergetics 1857.12 (2016), pp. 1860–1869. doi: https://doi.org/10.1016/j.bbabio.2016.09.003.
